# Human Metapneumovirus (hMPV) Infection and MPV467 Treatment in Immunocompromised Cotton Rats *Sigmodon hispidus*

**DOI:** 10.3390/v15020476

**Published:** 2023-02-09

**Authors:** Kevin C. Yim, Jarrod J. Mousa, Jorge C. G. Blanco, Sonnie Kim, Marina S. Boukhvalova

**Affiliations:** 1 Sigmovir Biosystems, Inc., 9610 Medical Center Drive, Suite 100, Rockville, MD 20850, USA; 2 Center for Vaccines and Immunology, Department of Infectious Diseases, College of Veterinary Medicine, University of Georgia, Athens, GA 30602, USA; 3 Department of Biochemistry and Molecular Biology, Franklin College of Arts and Sciences, University of Georgia, Athens, GA 30602, USA; 4 NIH/NIAID, Respiratory Diseases Branch, Division of Microbiology and Infectious Diseases, Rockville, MD 20852, USA

**Keywords:** hMPV, cotton rat, immunocompromised, MPV467

## Abstract

Human metapneumovirus (hMPV) is an important cause of respiratory disease in immunocompromised individuals, yet hMPV infection has not been modeled before in immunocompromised animals. In this work, cotton rats *S. hispidus* immunosuppressed by cyclophosphamide were infected with hMPV, and viral replication and pulmonary inflammation in these animals were compared to those in normal hMPV-infected *S. hispidus*. The efficacy of prophylactic and therapeutic administration of the anti-hMPV antibody MPV467 was also evaluated. Immunosuppressed animals had higher pulmonary and nasal titers of hMPV on day 5 post-infection compared to normal animals, and large amounts of hMPV were still present in the respiratory tract of immunosuppressed animals on days 7 and 9 post-infection, indicating prolonged viral replication. Immunosuppression was accompanied by reduced pulmonary histopathology in hMPV-infected cotton rats compared to normal animals; however, a delayed increase in pathology and pulmonary chemokine expression was seen in immunosuppressed cotton rats. Prophylactic and therapeutic MPV467 treatments protected both upper and lower respiratory tracts against hMPV infection. The lung pathology and pulmonary expression of IP-10 and MIP-1α mRNA were reduced by therapeutic MPV467 administration. These results indicate that immunosuppressed cotton rats represent a useful model for studying hMPV pathogenesis and for evaluating therapeutics that could alleviate hMPV-induced disease in immunocompromised subjects.

## 1. Introduction

Human metapneumovirus (hMPV) is a paramyxovirus first isolated in the Netherlands in 2001 from children with respiratory symptoms reminiscent of an RSV infection [1]. hMPV is a ubiquitous pathogen: 25% of children between the ages of 6 and 12 months are seropositive for hMPV, and essentially all children become seropositive by the age of 5 [1]. hMPV is detected in 4–16% of patients with acute respiratory tract infections [2,3,4]. The symptoms of hMPV range from mild upper respiratory tract infection to severe and life-threatening pneumonia and bronchiolitis [5]. Children younger than 6 months of age are about three times more likely to be hospitalized because of hMPV infection than children from 6 months to 5 years [6]. Other susceptible populations include children with underlying high-risk conditions [6], the elderly [7], and immunocompromised individuals [8,9].

Shortly after the isolation of hMPV, it became apparent that hMPV infection could cause severe disease in patients with hematologic malignancies and/or HSCT recipients [10,11]. A lower respiratory tract infection after hMPV exposure can develop in ~30–40% of adults and children with these conditions, with mortality reaching or exceeding 10–46% [10,11,12,13,14]. hMPV infection in immunocompromised individuals clinically resembles that caused by RSV [13], albeit co-infections between hMPV and RSV or hMPV and other viruses are rare [10,14,15]. The hMPV replication is significantly prolonged in immunocompromised individuals with severe disease [15], as well as in asymptomatic HSCT recipients [16,17]. The treatment approaches for HSCT recipients with hMPV LRTI infection include ribavirin alone, IVIG alone, or a combination of ribavirin and IVIG [14].

The cotton rat *Sigmodon hispidus* is an established model of respiratory virus infections, including those caused by RSV, influenza, adenoviruses, parainfluenza, rhinovirus, and enterovirus [18,19]. hMPV infection had been modeled in cotton rats *S. hispidus* shortly after the virus’ discovery [20,21,22,23]. The initial reports on *S. hispidus* susceptibility to hMPV infection ranged from it being the most permissive pulmonary replication model among multiple small animal models tested [21,24] to not supporting hMPV infection at all [25]. While these differences may be related to variations in animal manipulation and/or the specifics of the infectious material used, our experiments indicate that normal cotton rats are highly susceptible to infection with hMPV in both the upper and lower respiratory tract ([20,23], this work). hMPV infection has not been modeled before in immunocompromised cotton rats (or any other small animal model). In this work, we have developed an immunocompromised cotton rat model of hMPV infection to assess whether immunosuppression affects hMPV pathogenesis and if an anti-hMPV antiviral antibody can mitigate any adverse effects. A human monoclonal antibody MPV467 targeting a recently discovered pre-fusion-specific epitope on the hMPV F protein [26] was used for that purpose.

## 2. Materials and Methods

### 2.1. Reagents

Cyclophosphamide for injection (20 mg/mL USP, Sandoz Inc., Princeton, NJ, USA was obtained from Blue Door Pharma. MPV467, a human monoclonal antibody against the hMPV F protein, was produced as previously described [26].

### 2.2. Viruses and Viral Assays

The hMPV strain TN/94-49/A2 recovered from specimens collected in the Vanderbilt Vaccine Clinic (a kind gift of Dr. John Williams [10,27]) was grown on LLC-MK2 cells in a minimal essential medium supplemented with 0.2% glucose, 0.1% bovine serum albumin, 0.0002% trypsin, and 1% gentamicin. A single pool of hMPV (3 × 10^6^ pfu/mL) was used for the studies described herein.

### 2.3. Animals and Animal Studies

Inbred *S. hispidus* cotton rats were obtained from a colony maintained at Sigmovir Biosystems, Inc. Six to eight-week-old male and female cotton rats were used for the studies. Animals were housed in large polycarbonate cages and were fed a standard diet of rodent chow and water. The colony is monitored for antibodies, adventitious respiratory viruses, and other common rodent pathogens; however, no such antibodies were found (VRL Test 80221-RAT 1 Ab profile: *Carbacillus CARB*, *Toolan’s H-1 virus*, *Kilham Rat virus KRV*, *Mycoplasma pulmonis*, *Parvovirus generic*, *Pneumonia virus PVM*, *RCV/SDA*, *Sendai virus*). All studies were conducted under applicable laws and guidelines and after approval from Sigmovir Biosystems, Inc.’s Institutional Animal Care and Use Committee (IACUC). hMPV infection in immunocompromised animals and the efficacy of MPV467 treatment were verified in two consecutive experiments. The sample size of four to five animals per group was chosen based on the results of previous experiments and to allow the detection of statistically significant differences between the groups. A comparison between groups was run using Student’s *t*-test for unpaired data with unequal variance (KaleidaGraph). Unless indicated, samples were not blinded prior to analysis.

Immunosuppression was induced in cotton rats by repeated treatment with cyclophosphamide (CY) based on the method described before [28]. In brief, 50 mg/kg of CY solution was administered intramuscularly (i.m.) as 250 μL/100 g animal for 18 days on a Monday-Wednesday-Friday schedule. At the end of this period, whole blood was collected to verify the decline in total white blood cell and lymphocyte counts. CY treatment was continued until the end of the study. Twenty-one days after the start of CY treatment, animals were infected intranasally (i.n.) with hMPV (10^5^ PFU per animal). To quantify the hMPV load, groups of 5 animals were sacrificed on days 5 and 7 post-infection (the first study) or days 5 and 9 post-infection (the second study) for the collection of lungs and noses for viral quantification by plaque assay. A group of normal, age-matched cotton rats was infected with hMPV and sacrificed on days 5, 7, and 9 post-infection for the quantification of hMPV by plaque assay.

For the evaluation of dose-dependency for the prophylactic and therapeutic efficacy of MPV467, cotton rats immunosuppressed with cyclophosphamide were inoculated i.m. with 0.1, 1, or 10 mg/kg MPV467 one day before or three days after hMPV challenge. Control animals were treated with PBS (mock) one day before hMPV infection. Animals were sacrificed 5 days after hMPV challenge, and lungs and noses were collected for viral titration. For the analysis of the therapeutic efficacy of MPV467 at the time of delayed clearance (day 9), immunosuppressed animals were infected with hMPV, treated i.m. with 10 mg/kg MPV467 on day 3, and sacrificed on day 5 post-infection, or treated with MPV467 on days 3 and 7 and sacrificed on day 9 post-infection. Normal animals were treated with MPV467 3 days post-infection and were sacrificed on day 9. Lungs were collected for histopathology and qPCR analysis. Lungs and noses were collected for viral titration.

### 2.4. Viral Titration

Lung and nose homogenates were clarified by centrifugation and diluted in EMEM. Confluent LLC-MK-2 monolayers were infected in duplicates with diluted homogenates in 24-well plates. After one hour of incubation at 37 °C in a 5% CO_2_ incubator, the wells were overlaid with 0.75% methylcellulose medium. After 7 days of incubation, the overlays were removed, and the cells were fixed for one hour and air-dried for immuno-staining. Upon blocking the wells with 1% BSA in PBS, the mouse anti-hMPV-N-protein antibody at a 1:1000 dilution in 1% BSA was added to each well, followed by washes and then incubation with an HRP-conjugated Rabbit anti-mouse IgG diluted 1:1000 in 1% BSA. An AEC Chromogen detection solution was added to each well and incubated at room temperature for 2 h. Visible plaques were counted, and virus titers were expressed as plaque-forming units per gram of tissue. Viral titers were calculated as the geomean ± standard error (S.E.M.) for all animals in a group at a given time.

### 2.5. Lung Cytokine/Chemokine mRNA Analysis

The total RNA was extracted from homogenized lung tissue using the RNeasy purification kit (QIAGEN). One microgram of the total RNA was used to prepare cDNA using oligo dT primers and Super Script II RT (Invitrogen). Cotton rat cytokine/chemokine cDNA was analyzed by qPCR using the primers and conditions previously described [29,30,31]. The signal obtained for each analyzed gene was normalized to the level of β-actin (“housekeeping gene”) expressed in the corresponding organ. Cytokine/chemokine levels were expressed as the geometric mean ± S.E.M. for all animals in a group at a given time. Differences among groups were evaluated by the Student’s *t*-test of the summary data.

### 2.6. Histolopathology Analysis

Lungs were prepared for histopathology analysis as previously described and were scored blindly for peribronchiolitis (inflammatory cells around small airways), perivasculitis (inflammatory cells around small blood vessels), alveolitis (inflammatory cells within alveolar spaces), and interstitial pneumonitis (inflammatory cell infiltration and thickening of alveolar walls) [32]. Each parameter was scored on a 0–4 scale, with 0 = 0%, 1 = 5%, 2 = 25%, 3 = 75%, and 4 = 100% histological scores.

## 3. Results

### 3.1. Immunosuppression Results in Increased hMPV Replication and Delayed Viral Clearance in Cotton Rats

**Figure 1:**
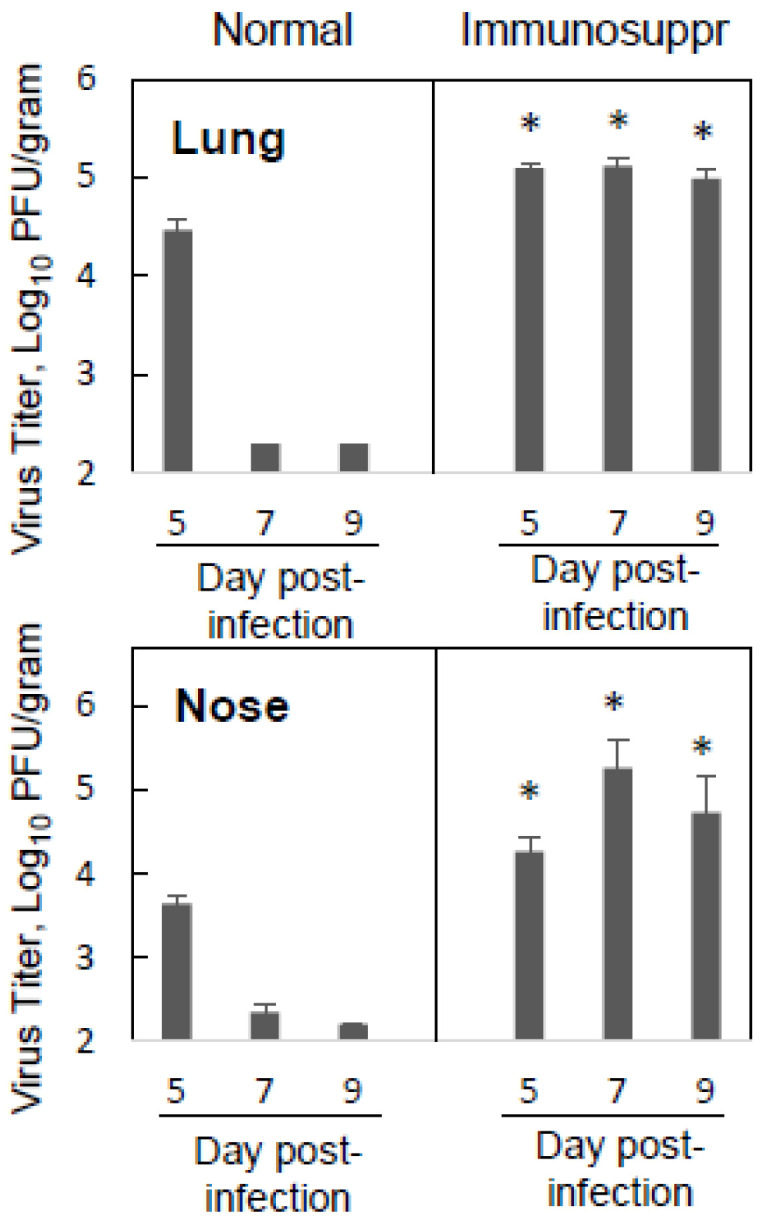
hMPV replication and clearance in immunosuppressed vs. normal cotton rats. Cotton rats immunosuppressed via repeated cyclophosphamide treatments (Immunosuppr) or normal cotton rats (Normal) were challenged with hMPV at 10^5^ PFU per animal. Five, seven, or nine days after infection, the animals were sacrificed, and lung and nose samples were collected for viral titration by plaque assay followed by immunostaining. Results represent the geomean ± S.E.M. for 4–10 animals per group (data combined from two studies). * *p* < 0.05 when compared to hMPV-infected normal animals sacrificed on the same day.

Immunosuppression can be associated with more severe disease and the prolonged replication of respiratory viruses in affected individuals [33,34,35]. To assess hMPV replication and clearance in cyclophosphamide-immunosuppressed cotton rats, animals were infected with 10^5^ PFU of hMPV, and viral replication was assessed at several time points after infection. Lung and nose samples were collected for viral titration on day 5 post-infection, the time of peak viral replication in normal cotton rats, on day 7, the time when the virus is cleared from the lungs of infected cotton rats, and on day 9, at an additional delayed time point. A comparison was made to normal age-matched animals infected in parallel with the immunosuppressed animals and sacrificed on the same days. As expected, in normal animals, hMPV was cleared from the lungs and essentially cleared from the nose of infected normal cotton rats by day 7 post-infection and was not detectable in either lower or upper respiratory tracts on day 9 (Figure 1). In contrast, immunosuppressed cotton rats showed high levels of hMPV in the lungs and nose on all three days. The amount of hMPV present in the lungs and nose of infected animals on day five post-infection was significantly higher than what was detected in normal cotton rats sacrificed at the same time. Overall, these results indicate that hMPV can replicate to higher titers in immunosuppressed cotton rats and that the clearance of the virus was significantly delayed by immunosuppression.

### 3.2. Prophylactic or Therapeutic Treatment with Anti-hMPV Antibody Reduces hMPV Load in Immunosuppressed Cotton Rats in a Dose-Dependent Manner

**Figure 2:**
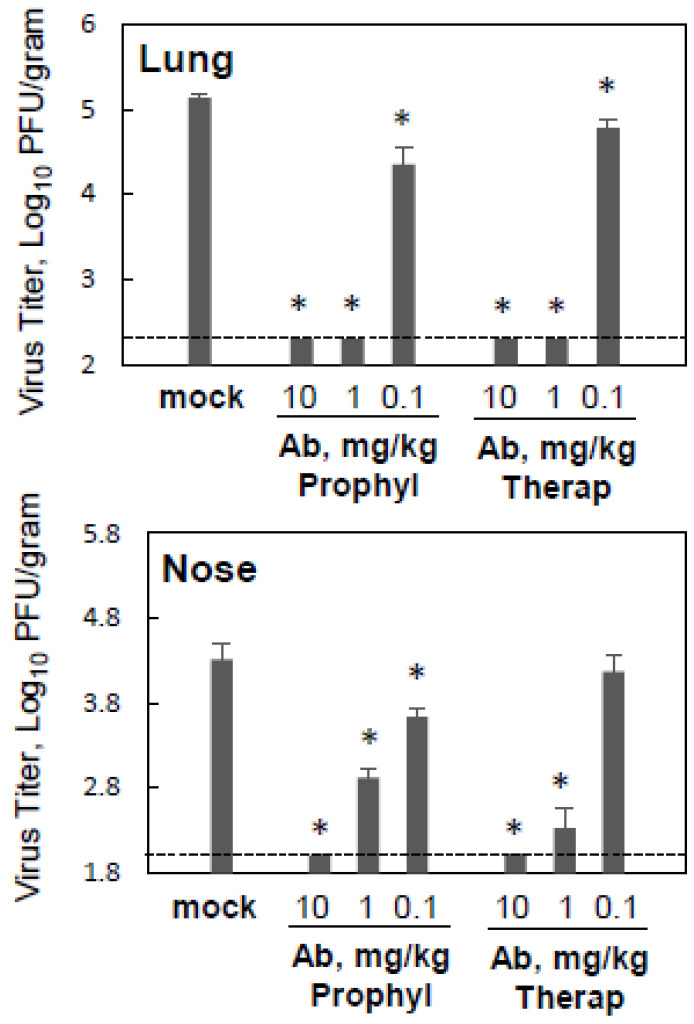
MPV467 prophylaxis and therapy of hMPV infection in immunosuppressed cotton rats: dose-dependency of the antiviral effect. Immunosuppressed *S. hispidus* were challenged with hMPV at 10^5^ PFU/animal. MPV467 treatment was administered intramuscularly as 0.1, 1, or 10 mg/kg one day before (Prophyl) or three days after (Therap) hMPV challenge. Five days after infection, animals were sacrificed and lung and nose samples were collected for hMPV quantification. Results represent the geomean ± S.E.M. for five animals per group. * *p* < 0.05 when compared to hMPV-infected mock-treated animals sacrificed on day five post-infection.

Monoclonal antibodies targeting viral surface proteins are among the most efficient therapeutics and prophylactics for viral infections used today [36]. Antibody MPV467 is a human monoclonal antibody against the hMPV fusion protein that was identified from a panel of human antibodies based on its binding avidity and strong neutralization potency towards hMPV [26]. The prophylactic and therapeutic efficacy of MPV467 was assessed in normal and immunosuppressed cotton rats infected with hMPV and sacrificed on day five post-infection (Figure 2). Three different doses of MPV467 were tested: 0.1, 1, and 10 mg/kg one day before or three days after infection. The results of the testing showed that both prophylactic and therapeutic treatments with MPV467 were effective at reducing hMPV replication in the lungs and noses of infected animals. The two highest doses of MPV467 that were tested, 10 and 1 mg/kg, given either before or after infection, completely protected the lungs of infected animals (undetectable viral replication). The lowest dose of MPV467 tested, 0.1 mg/kg, caused a modest but statistically significant reduction in lung hMPV replication, with the effect being slightly more pronounced for prophylactic treatment. In the nose, a significant reduction in the hMPV load was afforded by all three doses of MPV467 that were tested when administered prophylactically and by the two highest doses when given therapeutically. Overall, these results indicate that MPV467 has a strong dose-dependent antiviral efficacy in immunosuppressed cotton rats.

### 3.3. MPV467 Therapy Ameliorates Delayed hMPV Clearance in Immunosuppressed Cotton Rats

**Figure 3:**
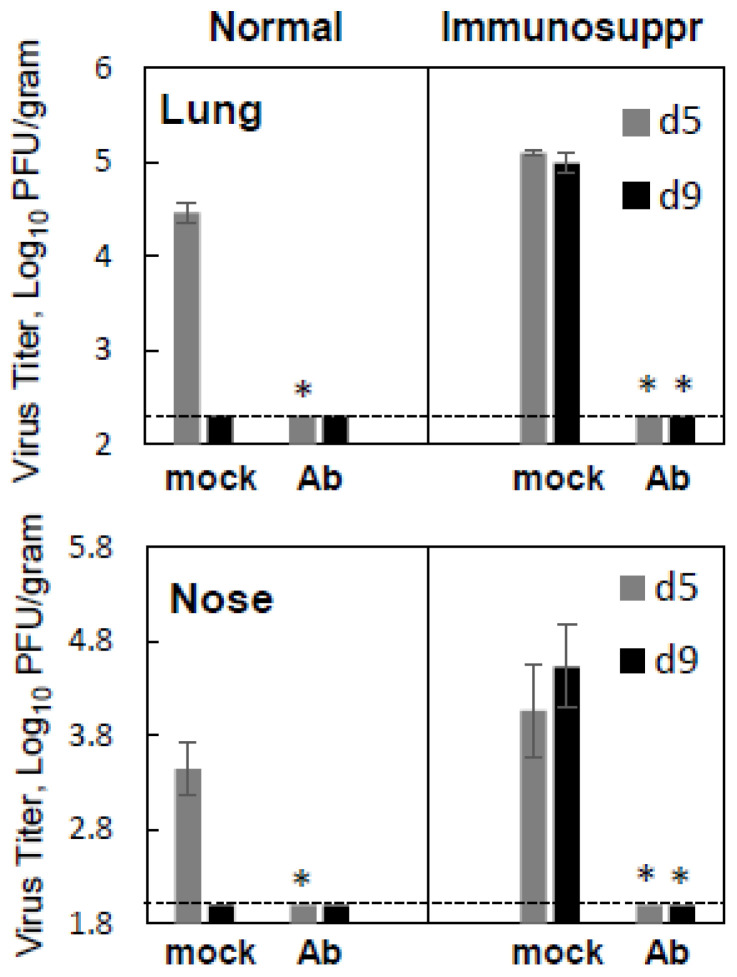
Effect of antibody therapy on delayed viral clearance in immunosuppressed cotton rats infected with hMPV. Immunosuppressed and normal *S. hispidus* were challenged with hMPV at 10^5^ PFU/animal and treated with MPV467 10 mg/kg on day three (normal) and days three and seven (immunosuppressed) after infection. Five or nine days after infection, the animals were sacrificed and lung and nose samples were collected for hMPV quantification. Results represent the geomean ± S.E.M. for 4–10 animals per group. * *p* < 0.05 when compared to hMPV-infected mock-treated animals sacrificed on the corresponding day.

Once the antiviral efficacy of MPV467 was ascertained in immunosuppressed cotton rats by analyzing samples collected at the peak time of viral replication in the lung, the question arose as to whether the therapeutic administration of antibodies would be able to combat delayed viral clearance in the model. To address this question, hMPV-infected immunosuppressed animals were treated with 10 mg/kg MPV467 three and seven days after infection and sacrificed on day nine post-infection for the analysis of the viral load in the lungs and nose. Normal cotton rats were infected and treated once on day three. Replication on day five was assessed again in hMPV-infected animals (normal and immunosuppressed) treated with 10 mg/kg MPV467 (or mock-treated) three days after infection. MPV467 was highly efficacious in normal cotton rats, reducing viral replication to undetectable levels in day five samples (Figure 3). MPV467 was also highly efficacious in immunosuppressed animals. Mock-treated hMPV-infected immunosuppressed animals had comparable amounts of hMPV that was detectable on days five and nine in the lungs and noses, while animals treated with MPV467 had no detectable hMPV in either the upper or lower respiratory tracts on both days of analysis.

### 3.4. Effect of Antibody Therapy on Lung Histopathology and Chemokine Expression in Immunosuppressed Cotton Rats

**Figure 4:**
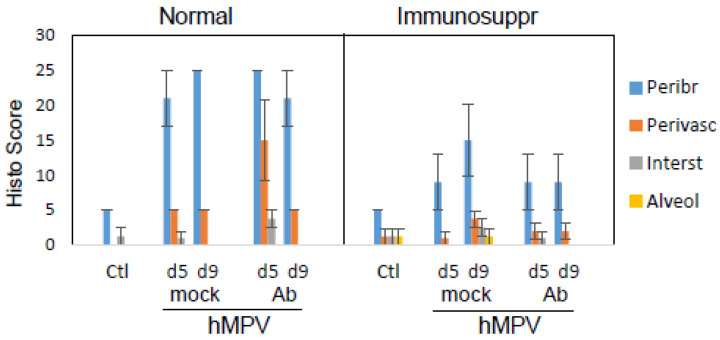
Effect of antibody therapy on lung histopathology in normal and immunosuppressed cotton rats infected with hMPV. Immunosuppressed and normal *S. hispidus* were challenged with hMPV at 10^5^ PFU/animal and treated with MPV467 as described in the legend of the previous figure. Pulmonary histopathology was evaluated in hematoxylin-eosin (H&E) slides in each of the following categories: peribronchiolitis (Peribr), perivasculitis (Perivasc), interstitial inflammation (Interst), and alveolitis (Alveol). Results represent the mean ± SE for 4–5 animals per group.

Pulmonary histopathology, one of the markers of the inflammatory response to respiratory infection in the cotton rat model, was used to assess the differences in lung response to hMPV in normal and immunosuppressed animals in the presence or absence of antibody treatment. Cotton rats were infected with hMPV and sacrificed on days five and nine post-infection for the analysis of peribronchiolitis, perivasculitis, interstitial inflammation, and alveolitis. Normal, hMPV-infected animals had a moderate level of pathology that was characterized predominantly by peribronchiolitis and some perivasculitis (Figure 4). The extent of pathology was largely comparable between days five and nine. Antibody treatments caused a moderate increase in perivasculitis in normal hMPV-infected cotton rats. Immunosuppressed cotton rats infected with hMPV developed reduced peribronchiolitis compared to normal animals. Pulmonary histopathology in hMPV-infected immunosuppressed animals was slightly higher on day nine compared to day five, with interstitial inflammation and alveolitis clearly evident. The effect of the therapeutic antibody treatment on lung pathology was evaluated for MPV467 administered as a 10 mg/kg dose. A decrease in pulmonary histopathology in hMPV-infected antibody-treated immunosuppressed animals on day nine post-infection was seen compared to hMPV-infected mock-treated immunosuppressed animals.

**Figure 5:**
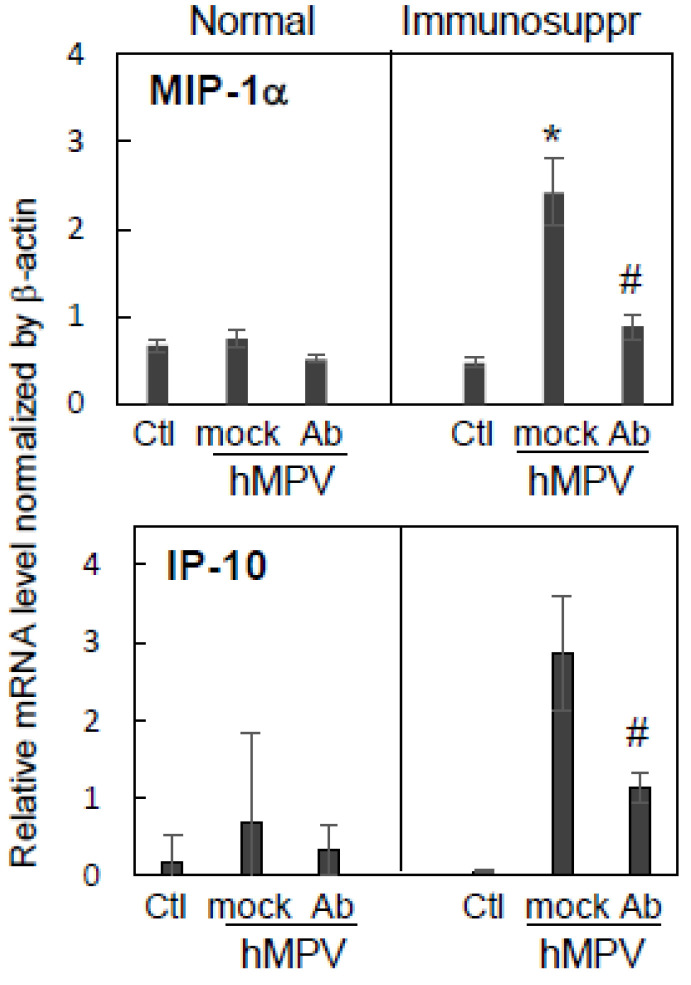
Effect of antibody therapy on pulmonary chemokine expression in normal and immunosuppressed cotton rats infected with hMPV. Immunosuppressed and normal *S. hispidus* were challenged with hMPV at 10^5^ PFU/animal, treated with 10 mg/kg MPV467 or PBS, and sacrificed 9 days post-infection. The expression of MIP-1α and IP-10 mRNA was quantified in the lung tissue by qPCR and normalized by the expression of β-actin in the corresponding organ. Results represent the mean ± SE for 4–5 animals per group. * *p* < 0.05 compared to normal animals uninfected (Ctl) or receiving the PBS (mock) or MPV467 (Ab) treatment. # *p* < 0.05 for antibody- vs. mock-treated animals.

The expression of lung cytokines/chemokines is another marker of the pulmonary inflammatory response to infections in the cotton rat model. To determine if a decrease in interstitial inflammation and alveolitis seen on day nine in immunosuppressed hMPV-infected animals treated with MPV467 was associated with altered pulmonary cytokine/chemokine expression, levels of pulmonary MIP-1α and IP-10 (mediators linked to lung injury and immune dysfunction [37,38,39,40]) were measured. Immunosuppressed animals experienced an elevated expression of MIP-1α and IP-10 mRNA compared to normal animals (Figure 5). The expression of both mediators in immunosuppressed animals was significantly reduced by antibody treatment.

## 4. Discussion

In this work, we describe for the first time an immunosuppressed animal model of hMPV infection. The model was based on a similar model for RSV, in which animals are immunosuppressed via repeated treatments with cyclophosphamide and subsequently infected with the virus [28,41,42]. Early studies on inducing immunosuppression in cotton rats called for a 50 mg/kg cyclophosphamide treatment to be administered intraperitoneally [41,42], which could be associated with complications, considering that animals had to receive three intraperitoneal injections per week for multiple weeks. More recent studies ([28], this work) demonstrated that the same dose of cyclophosphamide administered intramuscularly (a less traumatic approach) results in a similar immunosuppressive effect. A number of similarities were found between the immunosuppressed hMPV and RSV cotton rat models developed that way. Viral clearance was significantly affected in immunosuppressed cotton rats that were infected with either virus. In both models, virus-specific antibodies administered therapeutically were efficacious against both viral replication and pathology. Additionally, the pathology of the late infection (one week or more post-challenge) was elevated in immunosuppressed vs. normal animals, indicating that prolonged viral replication was accompanied by a long-lasting altered inflammatory response in the lung.

Normal (un-manipulated) cotton rats *S. hispidus* are susceptible to hMPV infection in the upper and lower respiratory tracts, with infection largely resolving within a week ([20,21], this work). This is different from persistent hMPV infection that has been reported for regular BALB/c mice, where hMPV replication can sometimes last from weeks to months [43,44,45]. The self-limiting hMPV infection in un-manipulated cotton rats resembled the self-limiting hMPV infection of healthy humans [46,47] and was a beneficial feature that allowed for the assessment of a potential viral clearance defect that could be caused by immunosuppression.

Similar to what has been reported for immunocompromised humans [15,16,17,48], hMPV replication was significantly prolonged in immunosuppressed cotton rats, confirming a delayed viral clearance under the conditions of suppressed immunity. Delayed viral clearance, in general, may impact lung function by a direct cytopathic effect of prolonged virus replication or by an indirect effect on lung inflammation. In this work, we noted that interstitial inflammation and alveolitis were slightly increased in hMPV-infected immunosuppressed cotton rats later in infection compared to the earlier time corresponding to peak viral replication in the lungs. Concurrently, the expression of pulmonary chemokines IP-10 and MIP-1α in immunosuppressed animals this late in infection surpassed that detected in normal cotton rats infected with hMPV. The increase in specific parameters of pulmonary inflammation later in the infection of immunosuppressed animals was similar to that seen before for RSV [28], with the exception that no cytokines/chemokines were measured for the RSV model and no detectable epithelial damage was seen in the lungs of hMPV-infected animals. Increased levels of IP-10 and MIP-1α late in hMPV infection in immunosuppressed animals may have a detrimental effect on the lung. Both molecules were shown to promote the development of lung injury of viral and non-viral origin through neutrophil-mediated mechanisms [37,38], and both molecules have been linked to the development of autoimmunity and pulmonary fibrosis [39,40].

Therapeutic administration of anti-hMPV antibody MPV467 resulted in the ablation of hMPV replication in immunosuppressed cotton rats, and it also reduced pulmonary pathology and chemokine expression in the lungs of immunosuppressed animals. This combined suppression of viral replication and pulmonary inflammation by a therapeutically administered antiviral antibody is similar to the effect seen in the RSV-infected immunosuppressed cotton rats treated with anti-RSV Ig [28]. For RSV, the ability of therapeutic antibody treatment to reduce lung inflammation in immunosuppressed animals contrasted with the lack of similar efficacy of antiviral antibodies when used alone in normal, non-immunosuppressed cotton rats [49,50] or in humans [51,52]. Because the inflammatory program already triggered by the viral infection cannot be stopped by an antiviral treatment alone, the addition of corticosteroids may be required to reduce pulmonary inflammation [49,50]. Albeit the studies to address a similar effect for hMPV still need to be designed, the data presented here show that MPV467, when administered after infection, reduces pulmonary inflammation in immunosuppressed but not normal animals, suggesting that a similar interplay between viral infection and inflammation may be in place for hMPV as well.

This study was associated with a number of limitations. First, immunosuppressed animals were analyzed for only 9 days after hMPV-infection. RSV-infected cotton rats immunosuppressed with cyclophosphamide could replicate RSV for two months if immunosuppression was continuously maintained [41]. A similar situation may be true for hMPV. However, the goal of this study was to create a concise experimental protocol for assessing the effects of immunosuppression on hMPV infection and for evaluating therapeutics in the model, and this objective was successfully met. The second limitation of this study was that a relatively low-titer hMPV stock was used to challenge the animals, which necessitated minimal dilution for infection. Moderate differences in viral replication between the lungs and noses of infected animals were detected with this viral stock, a difference that is less noticeable in the model when higher-titer stocks of hMPV are used (such stocks have been produced and tested since). In spite of these limitations, the studies reported here revealed the significant effect of immunosuppression on hMPV pathogenesis in cotton rats and demonstrated that therapeutic antivirals can ameliorate delayed viral clearance and pulmonary inflammation, with important implications for clinical disease in immunosuppressed individuals.

## Data Availability

All relevant raw data are available upon request (please contact the corresponding author).
